# Engineering of a probiotic yeast for the production and secretion of medium-chain fatty acids antagonistic to an opportunistic pathogen *Candida albicans*

**DOI:** 10.3389/fbioe.2023.1090501

**Published:** 2023-02-27

**Authors:** Hua Ling, Ruirui Liu, Qi Hui Sam, Haosheng Shen, Louis Yi Ann Chai, Matthew Wook Chang

**Affiliations:** ^1^ NUS Synthetic Biology for Clinical and Technological Innovation (SynCTI), National University of Singapore, Singapore, Singapore; ^2^ Synthetic Biology Translational Research Programme, Yong Loo Lin School of Medicine, National University of Singapore, Singapore, Singapore; ^3^ Department of Biochemistry, Yong Loo Lin School of Medicine, National University of Singapore, Singapore, Singapore; ^4^ Wilmar-NUS Corporate Laboratory (WIL@NUS), National University of Singapore, Singapore, Singapore; ^5^ Division of Infectious Diseases, Department of Medicine, National University Health System, Singapore, Singapore

**Keywords:** probiotic yeast, medium-chain fatty acids, *Candida albicans*, virulence, immunomodulation

## Abstract

*Candida albicans* is an opportunistic pathogen, with its infection as one of the causes of morbidity or mortality. Notably, the probiotic yeast *Saccharomyces cerevisiae* var. boulardii has shown the potential to fight against *Candida* infections. In this study, we aimed to engineer a commercial boulardii strain to produce medium-chain fatty acids (MCFAs) with antagonistic effects against *C. albicans*. First, we identified and characterized a boulardii strain and created its auxotrophic strain *Δura3*. Next, we constructed and expressed a heterologous MCFA biosynthetic pathway under the control of inducible and constitutive promoters. Aside from examining MCFA production and secretion, we confirmed MCFAs’ effects on *C. albicans’* anti-biofilm and anti-hyphal formations and the immunomodulatory effect of MCFA-containing supernatants on Caco-2 cells. We found that under constitutive promoters, the engineered boulardii strain constitutively produced and secreted a mixture of C6:0, C8:0, and C10:0. The secreted MCFAs then reduced biofilm and hyphal formations in *C. albicans* SC5314. We also confirmed that MCFAs upregulated the expression of virulence-related genes in SC5314. Furthermore, we found that the constitutively produced MCFAs in the supernatant induced the upregulation of immune response genes in Caco-2 cells co-cultured with SC5314, indicating MCFAs’ roles in immunomodulation. Overall, the engineered boulardii strain produced and secreted MCFAs, as well as demonstrated antagonistic effects against *C. albicans* SC5314 and immune-modulatory effects in Caco-2. To our knowledge, this represents the first study tackling the metabolic engineering of a commercial probiotic yeast strain to constitutively produce and secrete MCFAs showing anti-*Candida* effects. Our study forms the basis of the potential development of a live biotherapeutics probiotic yeast against *Candida* infections through metabolic engineering strategies.

## Introduction

Medium-chain fatty acids (MCFAs) are carboxylic acids with 6–12 carbons. MCFAs have demonstrated anti-pathogenic and immunomodulatory health effects, and are known to play a role in the regulation of body fat metabolism. Accordingly, MCFAs such as caprylic acid (C8:0), capric acid (C10:0), lauric acid (C12:0), or their combination with other compounds have been shown to inhibit pathogens like *Candida albicans* (CA) (Murzyn et al., 2010b; Lee et al., 2021), *Clostridium difficile* (Yang et al., 2017), *Listeria monocytogenes* (Hao et al., 2020), and *Salmonella* (Moschonas et al., 2012). In *Candida* spp, MCFAs may mimic the quorum sensing molecule farnesol that interferes with communications among fungal populations, leading to its potency against *Candida* (Lee et al., 2021). With regards to inflammation, C10:0 has been found to induce the upregulation of GPR84 and PPARγ and the downregulation of HIF-1α (Sam et al., 2021). In another study, C8:0 has been found to enhance inflammatory IL-8 secretion by >35% in human fetal intestinal epithelial cells (Andoh et al., 2000). Given MCFAs’ potency against pathogens and their roles in immunomodulation, MCFAs, their derivatives, and probiotics producing MCFAs have been widely used as anti-pathogenic agents and health supplements.

*Saccharomyces cerevisiae* var. boulardii (also named as *Saccharomyces boulardii*) is a probiotic yeast with versatile health effects. Not only are they anti-pathogenic and involved in immunomodulation, but they also promote the growth of beneficial gut microbiota—especially those associated with MCFAs produced and secreted by certain boulardii strains. For instance, Murzyn et al. found that a boulardii strain inhibits CA’s adhesion to intestinal cell lines and its extract reduces cytokine-induced inflammatory responses in Caco-2 cells as revealed by suppressed IL-8 expression (Murzyn et al., 2010a). Boulardii strains have also been found to diminish filamentation, biofilm formation, and CA translocation (Berg et al., 1993; Krasowska et al., 2009). Meanwhile, Tomicic and others reported how a boulardii strain significantly reduced the adherence ability of *Candida glabrata* ZIM2344 and ZIM2369 in a dose-dependent manner (Tomicic et al., 2016). The administration of the boulardii strains CNCM I-745, CNCM-I-1079, or CNCM-I-1079 + *Lactobacillus rhamnosus* GG was also found to expedite the reestablishment of a healthy microbiota after diarrheic dysbiosis (More and Swidsinski, 2015). In a piglet model, supplementation with a boulardii strain mafic-1701 was observed to improve feed conversion efficiency (Zhang et al., 2020). In some cases, boulardii strains can natively produce and secrete MCFAs represented by C10:0, which inhibits CA’s filamentous growth, adhesion, and biofilm formation (Murzyn et al., 2010b; Kunyeit et al., 2020; Duysburgh et al., 2021). Given these beneficial effects, boulardii strains have been widely used as a probiotic yeast to promote human health by inhibiting pathogens like *Candida* and modulating the gut microbiota. To date, several boulardii strains have been commercialized to promote human health.

*C. albicans* is a major opportunistic fungal pathogen. Clinically, current therapeutics for *Candida* infections mainly rely on the administration of anti-fungal drugs such as fluconazole, which has been implicated in multi-drug resistance (MDR). Notably, MDR associated with *Candida* has become a key clinical challenge in fighting fungal infections and a threat to public health (Berman and Krysan, 2020; Kunyeit et al., 2020). Consequently, alternative solutions are required to fight *Candida* infections more effectively. Given the proven anti-*Candida* effects of probiotic yeast and MCFAs, MCFA-producing boulardii strains could potentially offer an effective therapy against *Candida* infections. Nevertheless, MCFA production and secretion by wild type boulardii strains are strain- and condition-dependent, which could make it difficult to achieve desired anti-*Candida* effects. Therefore, a boulardii strain that stably produces and secretes MCFAs in a controllable manner would be ideal, and could be realized by expressing a heterologous MCFA biosynthetic pathway through synthetic biology-driven metabolic engineering strategies (Liu et al., 2016; Yu et al., 2016; Chen et al., 2018; Xia et al., 2019; Chen et al., 2020; Hossain et al., 2020).

In this study, we aimed to engineer a commercial probiotic yeast strain (*S. cerevisiae* var boulardii) to produce MCFAs with antagonistic effects against the opportunistic pathogen *C. albicans*. Briefly, we identified the ploidy of the boulardii strain, then constructed and expressed a heterologous MCFA biosynthetic pathway into *Δura3*. Next, we examined MCFA production and secretion, then confirmed MCFAs’ effects on anti-biofilm and anti-hyphal formations in *C. albicans* SC5314 cells and its immunomodulatory effects in the human epithelial cell line Caco-2. This represents the first study describing the metabolic engineering of a commercial probiotic yeast strain to produce MCFAs with anti-*Candida* effects. Our study provides useful insights into developing live biotherapeutics against *Candida* infections through metabolic pathway engineering.

## Materials and methods

Strains, growth conditions, plasmids, and chemicals. Strains and plasmids used are listed in Supplementary Table S1. *S. cerevisiae* S288C, *S. cerevisiae* var. boulardii, and *C. albicans* were grown in YPD (yeast extract 10 g/L, peptone 20 g/L, dextrose 20 g/L), YGD (yeast nitrogen base 6.7 g/L, dextrose 20 g/L, and yeast synthetic dropout medium supplements without uracil 1.92 g/L), or YRGD (yeast nitrogen base 6.7 g/L, D-raffinose 10 g/L, yeast synthetic dropout medium supplements without uracil 1.92 g/L, and D-(+)-galactose 20 g/L) at 30°C. *Escherichia coli* was grown in Luria–Bertani medium at 37°C. Hygromycin (200 mg/L) or ampicillin (100 mg/L) were added when applicable. DNA preparation kits were purchased from Qiagen (Germany), PCR reagents from Biorad (United States), restriction enzymes and DNA ligase from New England Biolabs (United Kingdom), and chemicals from Sigma-Aldrich (United States) unless specified.

Ploidy characterization by PCR. We isolated *S. cerevisiae* var. boulardii, designated as CLPY01, from Jarrow Formulas^®^ yeast probiotics (*Saccharomyces* Boulardii MOS). To verify the boulardii strain, we performed PCR using genomic DNA as a template along with intron splice site primers EI1 and LA1 (Fietto et al., 2004). We then compared the amplicon’s profile with that of *S. cerevisiae* S288C, boulardii U28, and M2 (Hamedi et al., 2013). To elucidate the boulardii’s ploidy after strain verification, we performed PCR using genomic DNA as a template by primers that pair with the MATa locus (MatF) or MATα locus (MatαF) and the reverse primer MatR (Supplementary Table S1).

Gene deletion. We amplified the *URA3* gene deletion cassette from pUG75, with this cassette containing a 45-bp upstream and downstream region flanking *URA3*’s coding sequence. The obtained gene deletion cassette was then transformed into CLPY01. To enable effective transformation, we used the following method for preparing competent cells: We prepared and diluted an overnight culture to OD_600_ 0.3 in fresh YPD medium, after which we grew it at 30°C and 225 rpm until the culture reached OD_600_ 1.6. Cells were collected and washed once with deionized H_2_O and then with an ice-chilled buffer (buffer 1: 1 M sorbitol + 1 mM CaCl_2_). Cells were suspended in 5 mL buffer (buffer 2: 0.1 M LiAc + 10 mM DTT) and incubated at 30°C and 225 rpm for 30 min. We then washed the cells once using 5 mL buffer 1 and then once using 1 mL 0.1 M LiAc. The cell pellet was then transformed using a LiAc/PEG-based method (Jung et al., 2021) followed by heat shock at 37°C for 1 h. Cells were recovered in YPD medium at 37°C for 1 h and spread on selective plates. We then screened colonies based on the presence of resistance to hygromycin and 5-fluoroorotic acid (5-FOA). The colonies showing resistance to both hygromycin and 5-FOA were further validated using YPD plates with and without uracil. Next, marker rescue was performed in a colony that grew only on a YPD plate (Ling et al., 2015). The resulting strain *Δura3* was named to CLPY02.

Gene cloning and overexpression. Genes including *hSFP*, *mhFAS,* and *rTEII* (sequences are shown in Supplementary Table S2) were synthesized by Life Technologies and cloned into pESC-URA standard protocols (Shao et al., 2009), resulting in the plasmid pESC-HR with *hSFP* under P_GAL10_ and *mhFAS-rTEII* under P_GAL1_. The recombinant plasmid pESC-HR was confirmed by sequencing and transformed into CLPY02 for MCFA production under D-galactose induction. Next, we replaced the promoters P_GAL10_ and P_GAL1_ to P_TDH3_ (for *hSFP* expression) and P_TEF1_ (for *mhFAS-rTEII* expression) in the opposite direction (sequences are shown in Supplementary Table S2), resulting in the plasmid pESC-gHR as confirmed by DNA sequencing. The used primers are listed in Supplementary Table S2.

Gene integration. We employed the DNA assembler method (Shao et al., 2009) to integrate the MCFA-biosynthetic pathway in pESC-gHR into CLPY02. Given the large size of the fragment, we divided the pathway into three fragments, including the terminator of *CYC1* and *ADH1* (HR1: 2965bp, HR2: 3016 bp, HR3: 4050 bp). HR3 was fused to a cassette expressing the hygromycin resistance gene (*HygR*) of pUG75 (HR3-HyrR) to facilitate colony screening with chromosomal integration. HR1, HR2, and HR3 contained 500-bp regions that overlapped with each other. HR1 and HR3-HyrR contained a 500-bp upstream or downstream region flanking the *URA3* locus. The agar plate with both hygromycin and uracil added was used to select colonies integrated with the gene cassette, and marker rescue was performed prior to the second round of chromosomal integration. To integrate another copy of the MCFA biosynthetic pathway, HR3 was fused to a cassette with corresponding overlapping regions that express the *URA3* of pUG72 (HR3-URA3) for chromosomal integration. HR1 and HR3-URA3 both contain a 500-bp upstream or downstream region flanking a *URA3* locus. The agar plate excluding uracil was used to select colonies integrated with the gene cassette. The resulting strain carrying two copies of the MCFA biosynthetic pathway was named CLPY04 and used for subsequent experiments.

Fatty acid extraction and detection. To measure fatty acids produced in engineered strains, single colonies were pre-cultured in appropriate media overnight. Cells were then inoculated into a fresh medium and incubated at 30°C and 225 rpm. Five milliliters of cells were harvested by centrifugation, and cell pellets were washed using deionized water. We added an internal standard heptadecanoic acid and lysed cells by adding 1.5 mL hydrochloride (HCl) and incubating for 1 h at 70°C. To derivatize fatty acids, we added 1.5 mL 10% HCl–methanol (v/v) into the lysate, followed by vortexing and incubation for 3 h at 62°C. After cooling down, fatty acid methyl esters (FAMEs) were extracted by adding 2 mL hexane and vortexing. Lastly, we centrifuged the sample and collected the upper hexane layer for GC analysis. For the detection of extracellular fatty acids, we added the internal standard and 10% HCl–methanol (v/v) into the supernatant and proceeded with fatty acid derivatization. GC analysis was performed following a previously reported method using an HP 7890 B GC system with an Agilent 5977 A MSD equipped with a HP-5MS column (Yu et al., 2016; Ng et al., 2020). GC peaks were identified by comparing the retention times and mass spectra of FAME standards. Data analysis was performed using the Agilent Enhanced Data Analysis software.

Biofilm and hyphal formation assays. *C. albicans* SC5314 was inoculated for overnight cultivation. The overnight culture was diluted to OD_600_ 0.1 in a microplate well containing supernatant (30%, v/v) + YPD (70%, v/v). The medium was replaced every 24 h. After incubation at 75 rpm and 37°C for 4 d, the biofilm was stained by crystal violet and measured at 590 nm. Under a microscope, hyphal formation was observed after 6 h incubation in supernatant (30%, v/v) + DMEM medium (DMEM1, without sodium bicarbonate and HEPES) (Sigma-Aldrich) (70%, v/v) at the appropriate pH. YRGD or YGD medium with the same volume as the supernatant was added as negative controls.

Co-culturing of Caco-2 and *C. albicans* SC5314. Caco-2 cells were seeded in a 6-well plate containing Gibco™ DMEM (DMEM2, with sodium bicarbonate and HEPES) + 10% fetal bovine serum with 5% CO_2_ supply at 37°C. The medium was changed every 24 h. On day 4, Caco-2 cells were pre-treated by DMEM1 for 1.5 h. Next, SC5314 cells (∼4 × 10^7^ CFU/mL) and MCFA-containing supernatant (30%, v/v) were added and incubated for 4 h. Caco-2 cells were collected and used for total RNA extraction as described below. YGD medium with the same volume as the supernatant was added as a negative control.

RNA extraction and real-time PCR (RT-PCR) analysis. The overnight culture of SC5314 was washed twice using customized DMEM and diluted in the appropriate medium to OD_600_ 2 in a 6-well plate. Cells were collected after incubation at 75 rpm and 37°C at the appropriate timepoints. Total RNA was extracted using an RNeasy Kit (Qiagen) following the provided manual. Complementary DNA synthesis and RT-PCR were performed to analyze the transcription levels of *BCR1*, *EFG1*, *HGC1*, *HWP1,* and *UME6* based on a reported method (Ling et al., 2015) using the 2^−ΔΔCT^ method. Gene expression was normalized to *ACT1*.

The total RNA of the co-cultured Caco-2 cells was extracted using a Trizol reagent (Hwang et al., 2021). RT-PCR was then performed to analyze the transcription levels of *IL-6*, *IL-8*, *LL-37*, *SA1009*, *CCL20*, and *hBD-3* using the 2^−ΔΔCT^ method. Gene expression was normalized to the *ACTB* gene.

## Results and discussion

### Development and characterization of the wild-type and auxotrophic boulardii strain

We firstly isolated the *S. cerevisiae* var. boulardii strain (named CLPY01 in this study) from a representative commercial yeast probiotic product (Jarrow Formulas) used to promote gut health.

**FIGURE 1:**
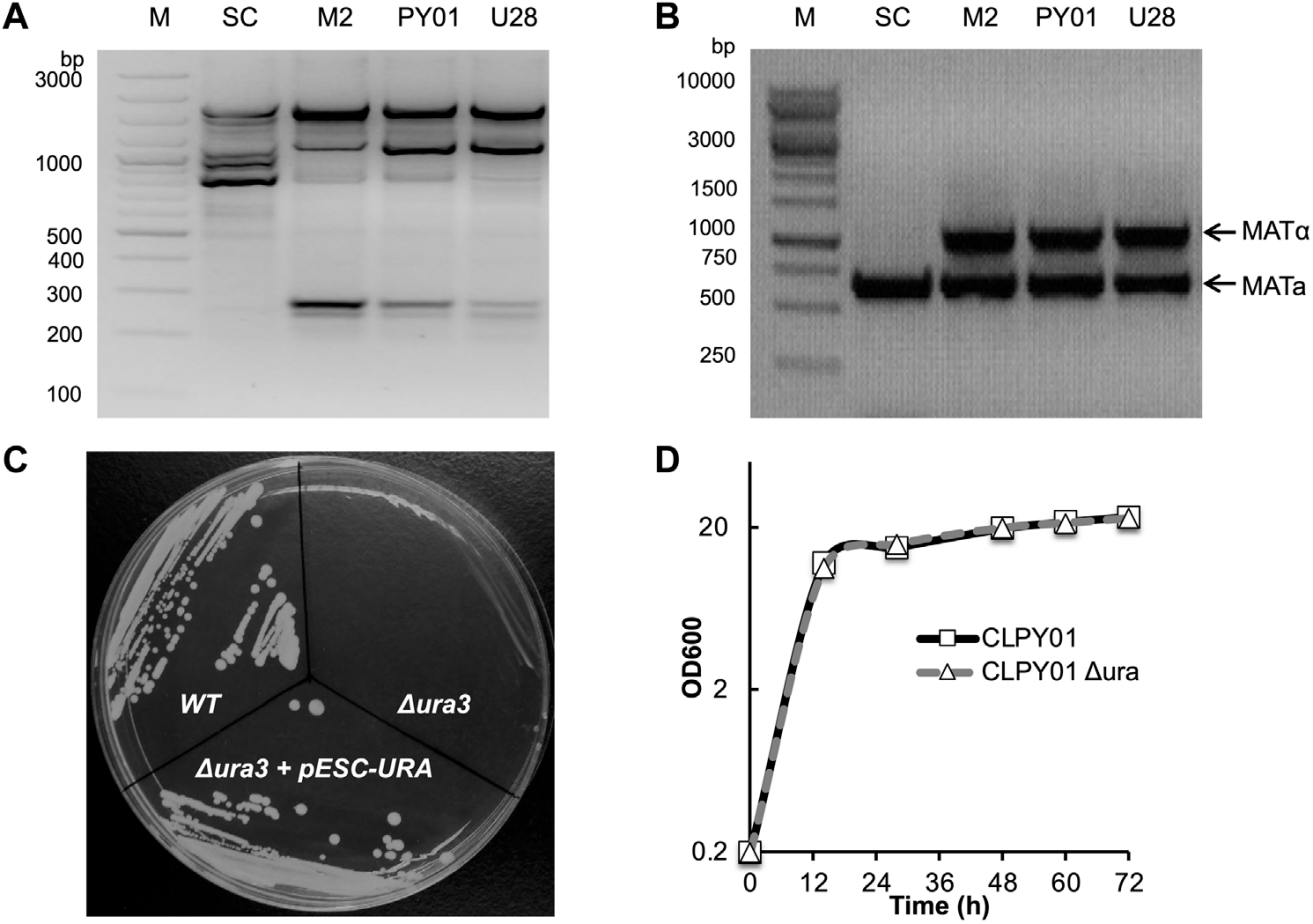
Identification of wild type and *Δura3* boulardii strains. **(A)**. PCR amplification of bands from genomic DNA. **(B)**. Ploidy. **(C)**. Phenotype identification of *URA3* gene deletion. *URA3* was deleted by using Cre-LoxP recombination. Cells were stroked onto an agar plate of minimal medium YGD without uracil. **(D)**. Growth pattern comparison of *S. boulardii* wild type and *Δura3* strains in YPD medium at 30°C. WT, CLPY01 wild type, *Δura3*, *URA3* gene deletion mutant, *Δura3*+pESC-Ura, *Δura3* harboring pESC-Ura.

To characterize the wild type strain CLPY01 (Genome Acc. No. JXBM00000000.1), we compared its genomic DNA amplicon profile and ploidy to other phylogenetically relative yeast strains. Figure 1A shows that strain CLPY01 has the same amplicon profiles as a known boulardii strain, namely Unique 28 (U28, Optibac Probiotics) and its mutant (M2) (Hamedi et al., 2013), both of which are distinct from *S. cerevisiae* S288C (SC). Next, we amplified the fragments located at the MATα and MATa sites in genome. Figure 1B shows that two fragments were amplified from CLPY01 along with U28 and M2, whereas only one fragment was amplified from SC. These results reveal that CLPY01 is a boulardii strain.

To create an auxotrophic strain suitable for metabolic engineering, we designed a hygromycin-resistant gene cassette and deleted the *URA3* gene by homologous recombination, resulting in the CLPY01 *Δura3* that we named CLPY02. After obtaining CLPY02, we identified the auxotrophic phenotype of *URA3* gene deletion by testing colony formation on uracil-free plates. Figure 1C shows that *S. boulardii* CLPY01 (WT) formed colonies on uracil-free plates, while CLPY02 did not form colonies, suggesting the deficiency of uracil biosynthesis in CLPY02 due to *URA3’s* absence. CLPY02 containing pESC-URA formed colonies, suggesting that *URA3*’s function is complemented by pESC-URA. This loss-and-gain function test confirms the successful deletion of *URA3* gene in CLPY01. Furthermore, we compared CLPY02’s growth pattern to its wild type strain CLPY01 in YPD medium. Figure 1D shows that CLPY02 had the same growth pattern as CLPY01, suggesting that there was no growth defect due to *URA3* deletion. Hence, the obtained auxotrophic boulardii strain CLPY02 was subsequently used for further experiments.

### Construction and inducible expression of a heterologous MCFA biosynthetic pathway in the boulardii strains

Despite several studies reporting the native production and secretion of MCFAs by *S. cerevisiae* var. boulardii strains, MCFA’s production level and composition remains subject to strain type and growing conditions (Murzyn et al., 2010b; Suchodolski et al., 2021). Therefore, we attempted to metabolically engineer CLPY02 to stably produce and secrete MCFAs by constructing and expressing a heterologous MCFA biosynthetic pathway.

**FIGURE 2:**
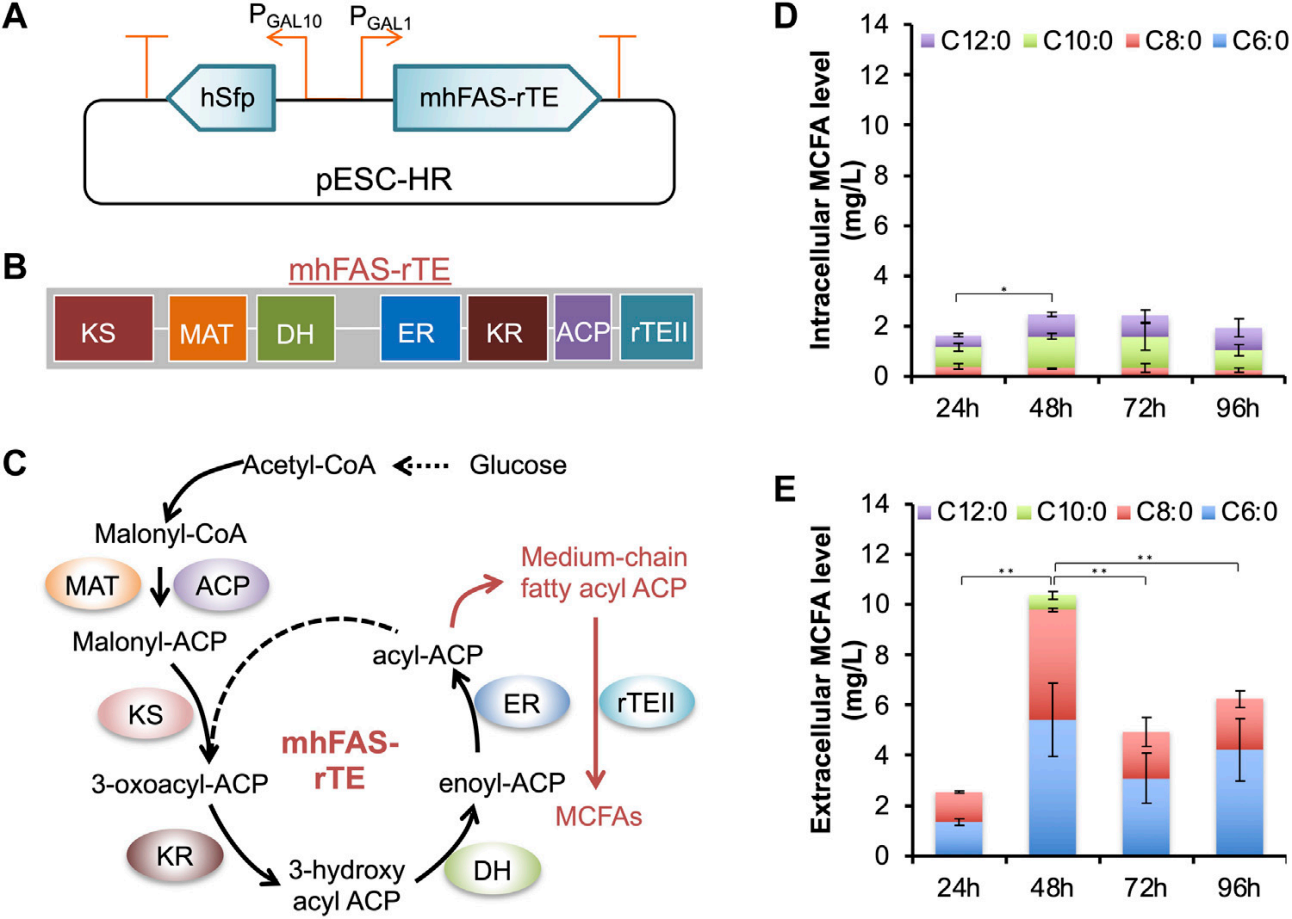
Plasmid-based inducible expression of an MCFA biosynthetic pathway and the analysis of MCFAs produced by the engineered strain CLPY03. **(A)**. Schematic of the recombinant plasmid pESC-HR harboring the MCFA biosynthetic pathway under the control of galactose-inducible promoters (P_GAL1_ and P_GAL10_). **(B)**. Organization of mhFAS-rTE domains. **(C)**. Schematic of the MCFA biosynthetic pathway. **(D)**. Measurement of the intracellular MCFAs. **(E)**. The time-course production level of MCFAs secreted into the medium. The data was obtained from at least three independent experiments. *, *p* < 0.05, **, *p* < 0.01 (Student’s t-test). *hSFP*, human phosphopantetheinyl transferase gene (encoding NP_056238.2), *mhFAS-rTEII*, a human fatty acid synthase mutant gene (*mhFAS*, encoding aa1-2,218 of NP_004095.4) fused with a rat thioesterase gene (*rTEII*, encoding NP_073196.1). KS, β-ketoacyl synthase, MAT, Malonyl-CoA-/acetyl-CoA-ACP-transacylase, DH, Dehydratase, ER, β-enyl reductase, KR, β-ketoacyl reductase, ACP, Acyl carrier protein.

In CLPY02, we cloned three genes for MCFA biosynthesis, including a mutant gene of *Homo sapiens* fatty acid synthase with its native thioesterase domain replaced by the *Rattus norvegicus* thioesterase II gene (mhFAS-rTEII) (Leber and Da Silva, 2014) and *H. sapiens* phosphopantetheinyl transferase gene (hSFP), resulting in pESC-HR (Table 1 and Supplementary Table S2). The expression of mhFAS-rTEII and hSFP are under the control of galactose-inducible promoters P_GAL1_ and P_GAL10_ (Figures 2A–C). After transforming pESC-HR into CLPY02, we measured the galactose-inducible production of MCFAs comprising of hexanoic acid (C6:0), octanoic acid (C8:0), decanoic acid (C10:0), and dodecanoic acid (C12:0). Figure 2D shows that the engineered strain CLPY03 produced about 2.4 mg/L MCFAs comprising of C8:0, C10:0, and C12:0 intracellularly. However, C6:0 could not be detected. Proportionally, C10:0 and C12:0 together accounted for over 60% of the produced MCFAs, whereas C8:0 had a lower abundance. Our time-course analysis shows the MCFA levels were the highest at 48 h and 72 h; Figure 2E shows that CLPY03 secreted a mixture of MCFAs into the extracellular environment, comprising of C6:0, C8:0, and C10:0 at up to 10.5 mg/L in total. Proportionally, C6:0 was slightly higher than C8:0, followed by C10:0 at 5% (at 48 h). C12:0 was not detectable. Time-course analysis shows that MCFA levels at 48 h was the highest. No MCFAs from CLPY02 carrying pESC-URA were detected in the supernatant. These results reveal that the engineered boulardii strain CLPY03 successfully produced and secreted the MCFA mixture (C6:0, C8:0 and C10:0) into the extracellular environment, up to 4.4-fold higher than the intracellular level.

**TABLE 1:** A list of the strains and plasmids used in this study.

Name	Description	References/Resources
Strains		
*Saccharomyces cerevisiae* S288C	A wild type strain	ATCC 204508
*S. cerevisiae* var. boulardii CLPY01	A wild type commercial probiotic yeast strain	Jarrow Formulas^®^
*S. cerevisiae* var. boulardii CLPY02	*Δura3* of CLPY01	This study
*S. cerevisiae* var. boulardii CLPY03	CLPY02 carrying the MCFA biosynthetic pathway under galactose inducible promoters cloned in pESC-URA	This study
*S. cerevisiae* var. boulardii CLPY04	CLPY02 chromosomally integrated with two copies of the MCFA biosynthetic pathway under constitutive promoters	This study
*S. cerevisiae* var. boulardii Unique 28	A wild type commercial probiotic yeast strain	Optibac
*S. cerevisiae* var. boulardii M2	An auxotrophic mutant obtained by UV mutagenesis	Hamedi et al. (2013)
Plasmids		
pESC-URA	An empty plasmid	This study
pESC-HR	pESC-URA carrying the MCFA biosynthetic pathway with genes encoding for human Type I FAS mutant (mhFAS), human phosphopantetheinyl transferase (hSfp), and rat thioesterase II (rTEII), induced by galactose	This study

There have been few reports on engineering boulardii strains for producing MCFAs. However, a recent study described how *S. cerevisiae* co-expression of a human FAS mutant, rTEII, and *Bacillus subtilis* Sfp allowed MCFA production and secretion (C6:0, C8:0 and C10:0). In another study, the co-expression of yeast FAS with site mutations and a short chain thioesterase resulted in the production and secretion of C6∼C12 fatty acids in *S. cerevisiae* (Gajewski et al., 2017). We noted that in these strains, C8:0 is the major MCFA secreted into the medium (Leber and Da Silva, 2014). Meanwhile, in the engineered boulardii strain CLPY03, the proportion of C8:0 and C10:0 is comparable. The variations among MCFA profiles may be due to differences in the enzyme’s chain-length specificity, host strains, and growth conditions. Given the proven anti-fungal effects of C8:0 and C10:0 (Krasowska et al., 2009; Murzyn et al., 2010b; Khalandi et al., 2020; Suchodolski et al., 2021), the secreted MCFAs containing C8:0 and C10:0 (48 h) may show antagonistic effects against *C. albicans*.

### Anti-*Candida* effects of MCFAs secreted by the engineered boulardii strain

To evaluate the anti-*Candida* effects of the secreted MCFAs by the engineered boulardii strain CLPY03, we analyzed the biofilm and hyphal formations of a representative *C. albicans* strain SC5314 in the presence of an MCFA-containing supernatant.

**FIGURE 3:**
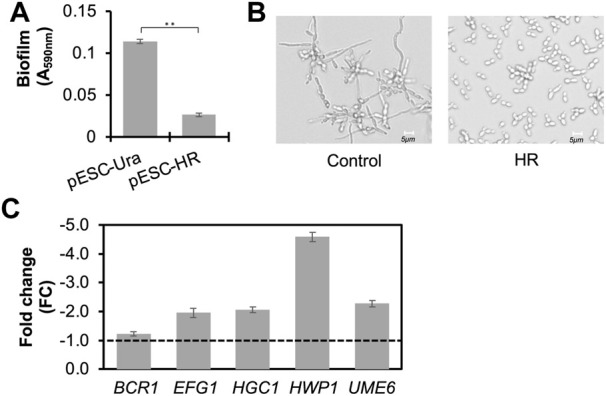
Functional analyses of the secreted MCFAs in the supernatant against biofilm and hyphal formations in *C. albicans* SC5314. **(A)**. Effect of the MCFA-containing supernatant from the engineered boulardii producing MCFAs (HR) on biofilm formation. Control, the strain carrying pESC-URA. **, *p* < 0.01 (Student’s t-test). **(B)**. Effect of MCFA-containing supernatant from the engineered boulardii producing MCFAs (HR) on hyphal formation at pH5.5. **(C)**. Real-time PCR analysis of the gene profile in *C. albicans* co-cultured with MCFA-containing supernatant from the engineered boulardii strain producing and secreting MCFAs (HR). The expression of genes related to biofilm and hyphal formations at 12 h was analyzed. The supernatant from the strain carrying pESC-URA was used as a control. Reference gene, *ACT1*. A dotted line indicates FC1.0, representing the fold change of genes from the control. Minus indicates the genes’ downregulation.

Figure 3A shows that adding 30% (v/v) of CLPY03’s supernatant at 48 h (pESC-HR) in YPD reduced biofilm formation by 4.3-fold over the control (CLPY02+pESC-URA). Under a microscope, we observed the formation of hyphae in the presence of the control supernatant. However, no obvious hypha formation was observed in the presence of CLPY03’s supernatant (HR) in a modified DMEM medium (Figure 3B). Given that biofilm formation and hyphal formation are related to *Candida*’s virulence, we were interested in exploring the expression profiles of virulence-relevant genes underlying HR-supernatant treatment in SC5314. Our real-time PCR analysis shows that *EFG1* (encoding a filament-specific regulator), *HGC1* (encoding a G1 cyclin-related protein), *HWP1* (encoding hyphal wall protein 1), and *UME6* (encoding a key filament-specific regulator) were downregulated by 2.0–4.6-fold (Figure 3C). These genes either positively regulate biofilm and hyphal formations or encode components of hyphae. In this study, their downregulation is in line with the reduced biofilm formation and hyphal formation in SC5314 with the HR-supernatant treatment. Notably, there was no change in the expression of *BCR1*, which encodes a biofilm formation regulator. These results suggest that the MCFA-containing supernatant of CLPY03 can reduce the expression of virulence-related genes, as well as biofilm and hyphal formations.

The aforementioned anti-*Candida* effects may be due to the MCFAs, proteins, or other metabolites produced and secreted by CLPY03. To confirm the cause of biofilm and hyphal formations, we spiked the MCFA mixture (C6:0, C8:0, and C10:0) with the same amount as the MCFAs secreted by CLPY03 (48 h) into the induction medium. Next, we compared the biofilm amount and hyphal formations of SC5314 with the spiked MCFAs to the induction medium (control). Supplementary Figure S1 shows that an MCFA spike-in reduced biofilm amount by 3.3-fold and inhibited hyphal formation. This result confirms that the secreted MCFAs in CLPY03 supernatant can reduce biofilm and hyphal formations in SC5314. We also noted that SC5314 treated with the CLPY02 supernatant had less amounts of biofilm (∼15-fold) than the induction medium, suggesting that other compounds (proteins, metabolites, etc.) in the CLPY02+pESC-URA supernatant may reveal anti-*Candida* effects, with future efforts required to elucidate those compounds.

### Constitutive expression of a heterologous MCFA biosynthetic pathway in the boulardii strain

In CLYP03, the inducible plasmid-based expression of the MCFA biosynthetic pathway resulted in the secretion of MCFAs with anti-*Candida* effects. However, MCFA production by CLPY03 is subject to the availability of galactose as an inducer, which is generally absent in *vivo* conditions and subject to the stability of the gene expression level and plasmid. To overcome this potential limitation, we expressed the MCFA biosynthetic pathway under constitutive promoters for the stable production and secretion of MCFAs.

**FIGURE 4:**
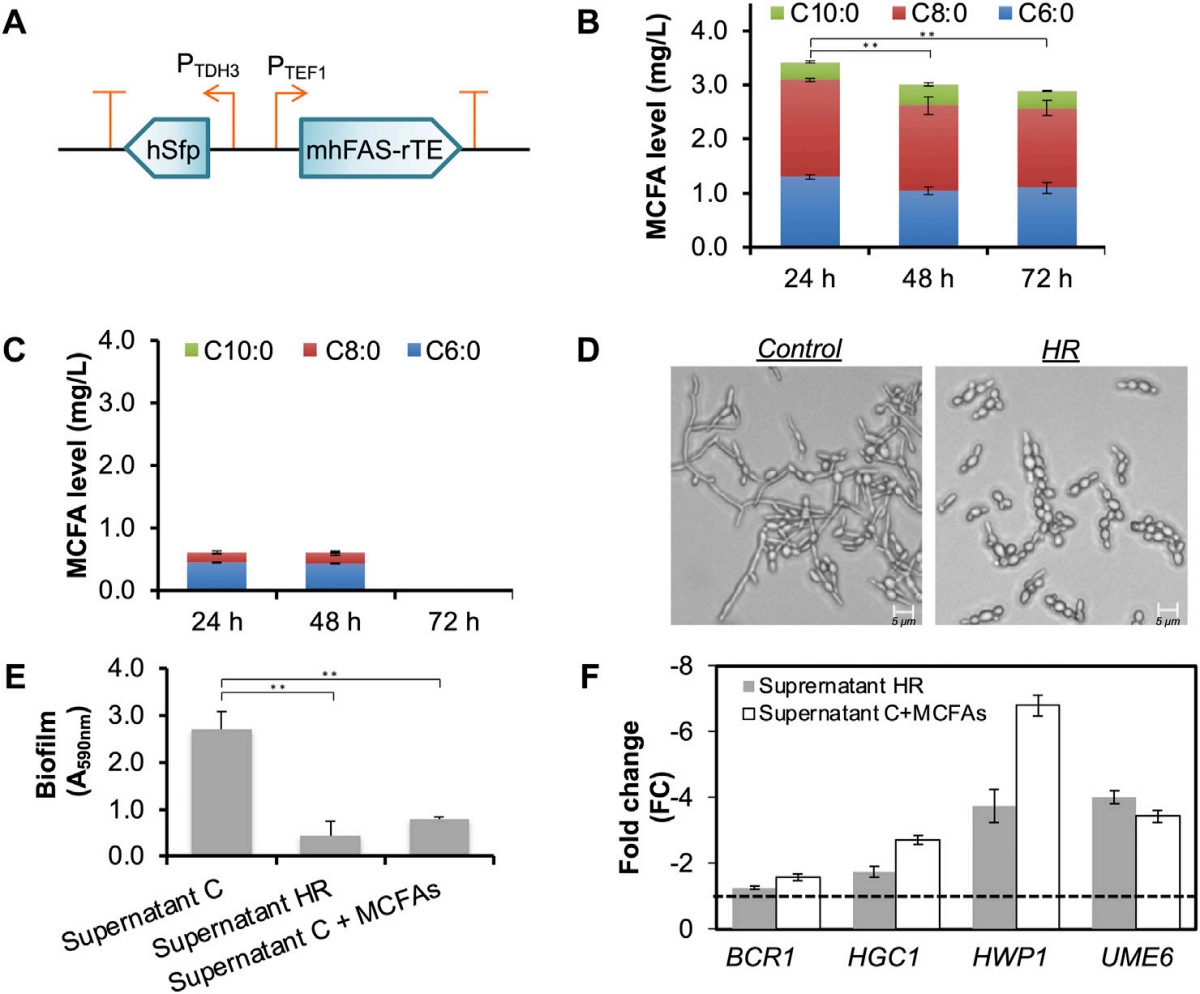
Chromosomal constitutive expression of an MCFA biosynthetic pathway and the functional analysis of the secreted MCFAs. **(A)**. Schematic of the recombinant plasmid pESC-HR harboring the MCFA biosynthetic pathway under the control of constitutive promoters (P_TEF1_ and P_TDH3_). **(B)**. The time-course production level of MCFAs secreted into the medium. The data was obtained from at least three independent experiments. **(C)**. MCFAs secreted by CLPY02 in YGD medium. **(D)**. Effect of the MCFA-containing supernatant from CLPY04 (HR) on hyphal formation at pH5.5. Control, the supernatant of CLPY01 which does not contain MCFAs. **(E)**. Effect of Supernatant HR on biofilm formations in the customized DMEM. Supernatant C, the supernatant of CLPY01. Supernatant C + MCFAs, Supernatant C with MCFA spike-in. **(F)**. Real-time PCR (RT-PCR) analysis of the gene profile in SC5314 co-cultured with Supernatant HR. Expression of genes related to biofilm and hyphal formation was analyzed. Supernatant C was used as a control. Reference gene, *ACT1*. A dotted line indicates FC1.0, representing the fold change (FC) of genes from the control. The pH of Supernatant C and Supernatant HR was adjusted to 5.5 prior to co-culturing. **, *p* < 0.01 (Student’s t-test).

To modify MCFA biosynthetic pathway expression, we replaced the inducible promoters (P_GAL1_ and P_GAL10_) with constitutive promoters (P_TEF1_ for mhFAS-rTEII, and P_TDH3_ for hSFP), and integrated each copy of the modified pathway with a *URA3* cassette and an *HygR* cassette (hygromycin resistance gene) respectively into the chromosome of CLPY02 at the original *URA3* locus (Figure 4A). We named the resulting strain CLPY04, which carries two copies of the MCFA biosynthetic pathway in the chromosome. Next, we measured the MCFAs secreted by CLPY04 expressing the MCFA biosynthetic pathway driven by constitutive promoters. Figure 4B shows that in the medium YGD, CLPY04 produced and secreted all three MCFAs (C6:0, C8:0 and C10:0) simultaneously over 72 h. The secreted MCFA amount by CLPY04 at 24 h (3.4 mg/L) was the highest, followed by 48 h (3.0 mg/L) and 72 h (2.9 mg/L). Proportionally, C8:0 was the highest MCFA secreted (∼52%), followed by C6:0 (∼37%), and C10:0 (∼11%). Overall, the composition and amount of the secreted MCFAs by CLPY04 remained almost the same, indicating that their stable production and secretion does not require a chemical inducer like galactose.

Interestingly, we detected a small amount of C6:0 and C8:0 (0.6 mg/L in total) in the supernatant of CLPY02+pESC-URA (Supernatant C), while no C10:0 was detected at 24 h and 48 h (Figure 4C). In contrast, CLPY02 under the induction medium (YRGD) did not secrete C6:0 nor C8:0 into the supernatant. Such differences in MCFA secretion by the same strain could be attributed to different growth conditions, consistent with the aforementioned case-dependence of MCFA production by the boulardii strains.

### Anti-*Candida* effects of MCFAs stably produced and secreted by the engineered boulardii strain

Upon the confirmation of MCFA production by CLPY04 under constitutive promoters, we next evaluated the anti-*Candida* effects of CLPY04’s MCFA-containing supernatant (24 h), including effects such as biofilm and hyphal formations.

To facilitate subsequent functional assays involving cell culture, we selected a commercial cell culture medium, Dulbecco’s Modified Eagle Medium (DMEM), to evaluate the anti-*Candida* effects of CLPY04’s supernatant (Supernatant HR). Surprisingly, we did not observe reduced biofilm and hyphal formations in the SC5314 in Supernatant HR (30% v/v) in DMEM. We noted that commercial DMEM maintains a high pH (pH7.5) due to a sodium bicarbonate buffer system. We hypothesized that the high pH maintained in commercial DMEM may have reduced or abolished the MCFAs’ anti-*Candida* effects. To confirm this possibility, we modified the pH of commercial DMEM by excluding sodium bicarbonate. Next, we adjusted CLPY01’s (Supernatant C) and Supernatant HR’s pH from 3.4 to 6.0. After adding them (30% v/v) to the customized DMEM, we observed hyphal formation, specifically finding that SC5314 formed hyphae when Supernatant C was at pH ≥ 3.4. We also found that hyphal formation increased along with pH, especially at pH ≥ 5.5. SC5314 formed hyphae with the Supernatant HR at pH6.0 (Figure 4D and Supplementary Figure S2). Our results validate our hypothesis on the influence of pH on anti-*Candida* activity, showing that a supernatant adjusted to pH5.5 is suitable for analyzing biofilm and hyphal formations. Hence, we decided to use supernatants adjusted to pH5.5 in the modified DMEM for the following analyses on biofilm and hyphal formations.

Figure 4E shows that adding 30% (v/v) of CLPY04’s supernatant (Supernatant HR, pH5.5) at 24 h into the modified DMEM reduced biofilm formation by 6.0-fold than Supernatant C. The spike-in of MCFAs into Supernatant C (Supernatant C + MCFAs) at an equal amount as Supernatant HR also reduced biofilm formation (3.5-fold) compared to Supernatant C. This result suggests that MCFAs secreted by CLPY04 reduce SC5314 biofilm formation. To further confirm biofilm and hyphal formations, we analyzed the expression of genes responsible for hyphal formation. Our real-time PCR analysis shows that *EFG1*, *HGC1*, *HWP1,* and *UME6* were downregulated by > 2.5-fold, and that there was no change of *BCR1* expression (Figure 4F), consistent with the reduced biofilm formation and hyphal formation in SC5314 respectively treated by CLPY04’s and CLPY03’s supernatants. Spiking-in MCFA to Supernatant C also downregulated the previously mentioned genes, except *BCR1*. These results suggest that the MCFA-containing supernatant of CLPY04 can reduce the expression of some virulence-related genes as well as biofilm and hyphal formations. The results also indicate anti-*Candida* effects like CLPY03 despite lower amounts of secreted MCFAs, especially of C10:0 in CLPY04 compared to CLPY03. Overall, the constitutive expression of the MCFA biosynthetic pathway has enabled the stable production and secretion of MCFAs reducing biofilm and hyphal formations in SC5314.

In the present study, the use of Supernatant HR comprising C6:0 (1.3 mg/L), C8:0 (1.8 mg/L) and C10:0 (0.3 mg/L) reduced both biofilm and hyphal formations in SC5314. Lee et al. reported that C8:0 and C10:0 at 1.0 mg/L respectively reduced biofilm formation in *C. albicans* DAY185 (Lee et al., 2021). In contrast, C6:0 at 37.8 mg/L or C8:0 at 17.9 mg/L does not affect the biofilm formation of SC5314 (Murzyn et al., 2010b). Given the relatively low concentration of the secreted MCFAs, the reduction of biofilm and hyphal formations by Supernatant HR might be attributed to the synergistic effects of C6:0, C8:0 and C10:0.

### Immunomodulation of MCFAs secreted by the engineered boulardii strain

To understand the role of the MCFA-containing supernatant of CLPY04 (Supernatant HR) in immunomodulation, we examined transcriptional levels of immunomodulation genes in a model human epithelial cell line Caco-2.

**FIGURE 5:**
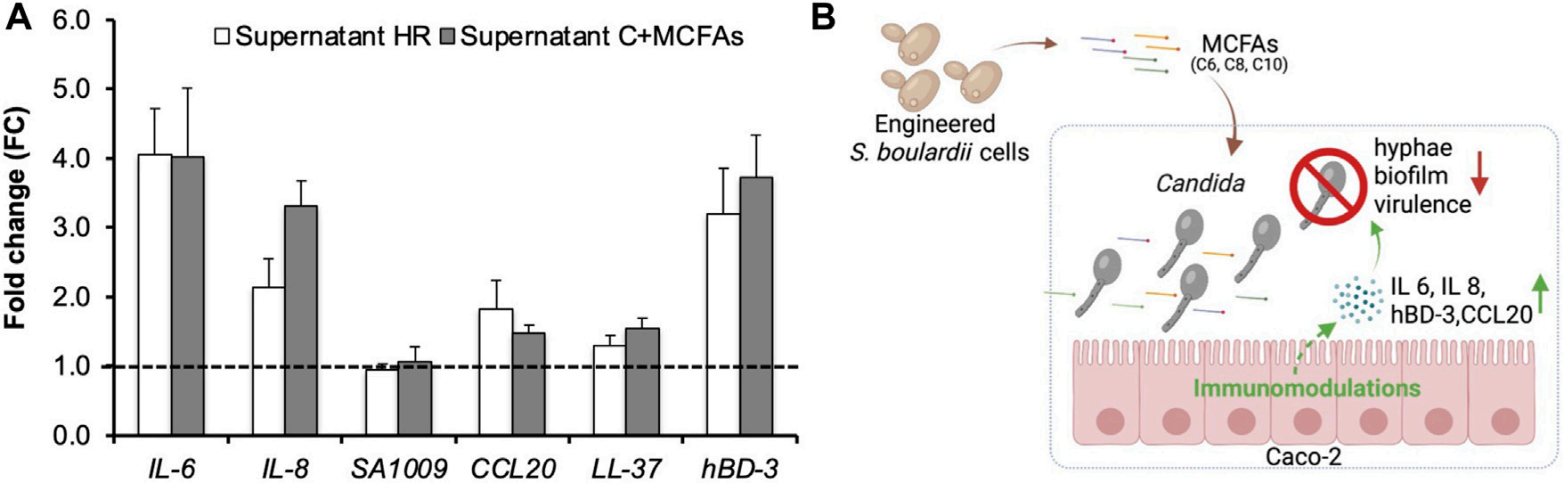
RT-PCR analysis of genes involved in immunomodulation in Caco-2 cells and the proposed mechanism of the MCFAs’ anti-*Candida* effects. Caco-2 cells were co-cultured with *C. albicans* and the MCFA-containing supernatant of CLPY04. **(A)**. Real-time PCR analysis. Expression of genes related to immunomodulation at 3.5 h was analyzed. The supernatant from the strain CLPY01 (Supernatant C) was used as a control. MCFAs were spiked into Supernatant C (Supernatant C + MCFAs) and used as a positive control. Reference gene, *ACT1*. A dotted line indicates FC1.0, representing the fold change of genes from the control. The pH of Supernatant C and Supernatant HR was adjusted to 5.5 prior to co-culturing. **(B)**. The proposed mechanism of anti-*Candida* effect by MCFAs. Reduction of *Candida*’s hyphae and biofilm formations, and virulence-related genes as well as the upregulation of IL-6, IL-8, human beta-defensin 3 (hBD-3), and CCL20 genes are indicated by arrows. The box in blue indicates the co-culturing of *C. albicans* cells, MCFA-containing supernatant, and Caco-2 cells.

Firstly, in a trans-well, we co-cultured Caco-2 cells with live SC5314 cells and Supernatant HR (30%, v/v, pH5.5) in the modified DMEM lacking the pH buffering capacity. Next, we analyzed the transcriptional level of immunomodulatory genes *via* real-time PCR. Figure 5A shows that the supplement of Supernatant HR upregulated the expression of *IL-6* (FC4.1), *IL-8* (FC2.1), *CCL20* (FC1.8), and *hBD-3* (FC3.2) in Caco-2 cells in the presence of *C. albicans* SC5314 cells. *IL-6* gene encodes Interleukin-6 (IL-6), while *IL-8* gene encodes Interleukin-8 (IL-8), both of which are pro-inflammatory cytokines produced in response to pathogenic infections. The two cytokines contribute to host defense by stimulating an immune response (Tanaka et al., 2014; D’enfert et al., 2021; Sam et al., 2021). The *hBD-3* gene encodes an anti-microbial peptide β-Defensin 3 (hBD-3) that shows a wide range of immunomodulatory functions and protects the gut during *Candida* infections (Fusco et al., 2021). *CCL20* gene encodes the chemokine ligand-20, which plays a role in attracting immune cells to infectious sites during the early stages of infection (Ma’ayeh et al., 2018). The upregulation of these genes indicates the immunomodulatory effects of the Supernatant HR in Caco-2 cells in response to the *Candida* infections.

To confirm the MCFAs’ immunomodulatory effects, we spiked commercial MCFAs into CLPY02’s supernatant with an equal amount of Supernatant HR and compared the gene profiles in Caco-2 cells co-cultured with SC5314. Figure 5A shows that MCFA spike-in (Supernatant C + MCFAs) resulted in the upregulation of *IL-6* (FC4.0), *IL-8* (FC3.3), *CCL20* (FC1.5) and *hBD-3* (FC3.7), sharing a similar induction trend to Supernatant HR. This result confirms that MCFAs present in the Supernatant HR can upregulate these genes. The different expression between Supernatant C + MCFAs and Supernatant HR might be due to other compounds in the supernatants. Hence, the MCFAs secreted by the engineered boulardii strain CLPY04 can alter immunomodulation in Caco-2, and likely trigger host defenses against *C. albicans* infections. Given the relatively low concentration of MCFAs in the supernatant, the upregulation of these genes by Supernatant HR might be attributed to the synergistic effects of C6:0, C8:0 and C10:0. The hyphal development of *C. albicans* might also impact cytokine gene expression. These results demonstrate the potential of applying CLPY04 as a therapeutic agent against *Candida* infections.

## Conclusion

In this study, we characterized and metabolically engineered a commercial probiotic yeast *S. cerevisiae* var. boulardii strain for antagonistic effects against the opportunistic pathogen *C. albicans*. The engineered boulardii strain was shown to stably produce and secrete an MCFA mixture comprising C6:0, C8:0, and C10:0. We found that the secreted MCFAs showed antagonistic effects to the opportunistic pathogen *C. albicans*, as demonstrated by reduced biofilm and hyphal formations. Through RT-PCR analysis, we confirmed that the secreted MCFAs downregulated the expression of virulence-related genes in *C. albicans*. Furthermore, we found that MCFAs induced the upregulation of immune response genes in Caco-2 cells co-cultured with *C. albicans*, indicating MCFAs’ roles in immunomodulation (Figure 5B). To our knowledge, our study represents the first study tackling the metabolic engineering of a commercial probiotic yeast for anti-*Candida* applications. Overall, our findings provide the basis for potentially developing a live biotherapeutics probiotic yeast against fungal infections such as *Candida* strains.

The engineered boulardii strain was proven to produce and secrete MCFAs showing antagonistic effects against *C. albicans*. We confirmed the antagonistic effects through *in vitro* assays by supplying the MCFA-containing supernatant from the engineered boulardii strain into the co-culturing system. In the future, we could further investigate the attribution of individual MCFAs, as well as specific combinations of different MCFAs at varying doses to the downregulation of virulence-related genes in *Candida* strains. Future efforts could also consider further optimizing the engineered strains and validating their antagonistic effects. For instance, native fatty acid synthesis could be tuned and blocked, while other competing pathways could be removed to increase the metabolic fluxes towards MCFA biosynthesis. We observed a slower growth and lower cell density of CLPY04 compared to the control CLPY02 (Supplementary Figure S3), suggesting a need to further improve cell growth. Moreover, it would be interesting to determine CLPY04’s genetic stability. Given the boulardii strains’ relatively low engineering efficiency, strain engineering could be facilitated by using synthetic biology toolkits such as CRISPR-based genome editing (Liu et al., 2016) and an optimal method with higher transformation efficiency. The engineered MCFA-producing strain could be directly co-cultured to validate its antagonistic effects against *Candida*. In addition, the efficacy of the engineered boulardii strain could be validated using an *in vivo* model.

## Data Availability

The original contributions presented in the study are included in the article/Supplementary Material, further inquiries can be directed to the corresponding author.
