# Supplementary material for: Engineering of a probiotic yeast for the production and secretion of medium-chain fatty acids antagonistic to an opportunistic pathogen Candida albicans
Source: Front Bioeng Biotechnol. 2023 Feb 27;11:1090501. doi: 10.3389/fbioe.2023.1090501 (PMC10008859; doi:10.3389/fbioe.2023.1090501)
Supplement: Supplementary file 1 [file DataSheet1.pdf]

Engineering of a probiotic yeast for the production and secretion of medium-chain fatty acids antagonistic to an opportunistic pathogen *Candida albicans*

Hua Ling[1,2,3,4*#], Ruirui Liu[1,2,3,4*], Qi Hui Sam[1,2,3], Haosheng Shen[1,2,3,4], Louis Yi Ann Chai[1,2,5], Matthew Wook Chang[1,2,3,4 **]

[1] NUS Synthetic Biology for Clinical and Technological Innovation (SynCTI), National University of Singapore, Singapore 117456, Singapore;
[2] Synthetic Biology Translational Research Programme, Yong Loo Lin School of Medicine, National University of Singapore, Singapore 117456, Singapore;
[3] Department of Biochemistry, Yong Loo Lin School of Medicine, National University of Singapore, Singapore 117596, Singapore;
[4] Wilmar-NUS Corporate Laboratory (WIL@NUS), National University of Singapore, Singapore 117599, Singapore;
[5] Division of Infectious Diseases, Department of Medicine, National University Health System, Singapore 119228, Singapore.

Present addresses
[#] HL: Wilmar Innovation Centre, Wilmar International Limited, 28 Biopolis Road, Singapore 138568

* The authors contributed equally to this work.

** Corresponding author: Matthew Wook Chang (bchcmw@nus.edu.sg)

Supplementary Information

Table S1. Oligos used in this study

Table S2. DNA sequences used for MCFA biosynthesis

Figure S1. Functional analyses of commercial MCFAs against biofilm and hyphal formation in *C. albicans* SC5314

Figure S2. Hyphal formation of *C. albicans 5314* treated by the CLPY04 supernatant and CLPY01 supernatant under different pH conditions

Figure S3. Growth patterns of CLPY04 and CLPY02 in YGD medium containing uracil

Table S1. Oligos used in this study

Name	Sequence (5'→3')
Boulardii strain characterization	
EI1	CTGGCTTGGTGTATGT
LA1	GCGATCGGTGTACTAAC
MatF	GAGAAGAGCCCAAAGGAAAATCAT
MatαF	GTCTAGTATGCTGGATTTAAACTCA
MatR	AGTCACATCAAGATCGTTTATGG
Gene deletion and integration	
pUG72-UraF	TATGGGAGGAAGAGAAGAAG
HygBR	GCAGCGATCGCATCCATTG
URA3uF	TAGAACGCGGCCGCGAACAAACACCAGAGTCAAA
URA3uR	TTTGGGACGCTCGAAGATGATTTATCTTCGTTTCCTGC
URA3dF	AGATCCACTAGTTGCATGTATACTAAACTCACAA
URA3dR	ATGCAGGTTAACGTTTTGTTCTTGGAAACGCT
HR1F	GCAGGAAACGAAGATAAATCATCTTCGAGCGTCCCAAAA
HR1R	GTCGACCTGCAGGGTGGTTGAGTATTTGGTCT
HR2F	TAGAACGCGGCCGCTTGTTGCTGTTTATTGACCA
HR2R	GTCGACCTGCAGTCTTCATCAGCCAATATACCC
HR3F	TAGAACGCGGCCGCAACATGCCTTCCTTGAAGAT
HR3R	GTCGACCTGCAGGAGCGACCTCATGCTATAC
Tef1p-Tdh3pF1	AATTAAGAGCTCAGTTTATCATTATCAATACT
Sfp-Tdh3pR1	GTCCATGTAGATACCGTATATCTTCATTTTGTTTGTTTATGT GTGTTT
Tdh3p-SfpF1	AAACACACATAAACAAACAAAATGAAGATATACGGTATCT ACATGGAC
Cyc1t-SfpR2	CTAACTCCTTCCTTTTCGGTTAGAGCGGATCTTAGCTAGCC GCGGTACCA
Tdh3p-Tef1pF2	AGATCTGAGCTCATAGCTTCAAAATGTTTCTA
hFAS-rTE-Tef1pR2	ACGATAAGATCTTTCCATTTTGTAATTAAAACTTAGATTA
RT-PCR	
CaBCR1-F	AATAAACTGGATCCTCCACC
CaBCR1-R	GTAGCATTAGTAGTAGCCGT
CaEFG1-F	ATTACACAAGTGCTCCTAGT
CaEFG1-R	GGATAGGTACTGCTTGTTGA
qCaHGC1-F	AATGGTAGTGTTATGAGTGGA
qCaHGC1-R	GGAGAATCATTTTCACTAATAGGT
CaHWP1-F	CACAGGTAGACGGTCAAGGT
CaHWP1-R	AAGGTTCTTCCTGCTGTTGT
CaUME6-F	TTACACCCGATTCAACTTCT
CaUME6-R	GTACTGGTGAAGACGAACTA
CaACT1-F	TTTCATCTTCTGTATCAGAGGAACTTATTT
CaACT1-R	ATGGGATGAATCATCAAACAAGAG
IL-6-F	CTATGGAAGGGTCCTACTCA
IL-6-R	GGCTATGGGATGATGTTCAA
IL-8-F	TGCGCCAACACAGAAATTAT
IL-8-R	CTGTAATCCTAACACCTGGA
LL-37-F	AGAGAAGATTGGCAAAGAGTT
LL-37-R	GGGTACAAGATTCCGCAAAA
SA1009-F	CAGTTTGGGTTGACAAGGGA

SA1009-R	ATCTTCCCCTTGATTGCTGTA
CCL20-F	TCTCCTCAGGTATGTTACACT
CCL20-R	ACTCCTCTTTCCTCAAACATT
HBD3-F	GAGTCAGTATTTCTCCATCCT
HBD3-R	AGTTCCCATACAACAAACCT
ACTB-F	CTGGAACGGTGAAGGTGACA
ACTB-R	AAGGGACTTCCTGTAACAATGCA

Table S2. DNA sequences used for MCFA biosynthesis

Name	Nucleotide sequence
hSFP	ATGGTTTTCCCAGCTAAGAGATTCTGTTTGGTTCCATCTATGGAAGGT<br>GTTAGATGGGCTTTTTCTTGTGGTACTTGGTTGCCATCAAGAGCTGAA<br>TGGTTGTTGGCTGTTAGATCAATTCAACCTGAAGAAAAAGAAAGAAT<br>CGGTCAATTCGTTTTCGCTAGAGATGCTAAAGCTGCTATGGCTGGTA<br>GATTGATGATTAGAAAATTGGTCGCCGAAAAGTTGAACATCCCTTGG<br>AATCATATCAGATTGCAAAGAACTGCTAAGGGTAAACCAGTTTTGGC<br>TAAGGATTCTTCTAATCCATACCCAAACTTCAACTTCAACATCTCCCA<br>TCAAGGTGATTATGCTGTTTTGGCTGCTGAACCAGAATTGCAAGTTG<br>GTATCGATATTATGAAGACCTCATTTCCAGGTAGAGGTTCCATTCCA<br>GAATTTTTCCATATCATGAAGAGAAAGTTCACCAACAAAGAATGGGA<br>AACCATCAGATCCTTTAAGGATGAATGGACCCAATTAGACATGTTCT<br>ACAGAAACTGGGCCTTGAAAGAATCTTTTATCAAGGCTATTGGTGTC<br>GGTTTGGGTTTCGAATTACAAAGATTGGAATTTGATTTGTCCCCATTG<br>AACTTGGACATCGGTCAAGTTTACAAAGAAACCAGATTATTCTTGGA<br>TGGTGAAGAAGAAAAAGAATGGGCCTTCGAAGAATCCAAGATCGAT<br>GAACATCATTTCGTTGCTGTTGCTTTGAGAAAACCAGATGGTTCAAG<br>ACATCAAGATGTTCCATCTCAAGATGATTCTAAGCCAACTCAAAGAC<br>AATTCACCATCTTGAACTTCAACGACTTGATGTCATCTGCTGTTCCAA<br>TGACTCCAGAAGATCCATCTTTTTGGGATTGCTTTTGTTTCACCGAAG<br>AAATCCCAATCAGAAACGGTACAAAATCCCATCATCATCACCATCAC<br>TGA
P_{TDH3}-P_{TEF1}	TTTGTTTGTTTATGTGTGTTTATTCGAAACTAAGTTCTTGGTGTTTTAA<br>AACTAAAAAAAAGACTAACTATAAAAGTAGAATTTAAGAAGTTTAA<br>GAAATAGATTTACAGAATTACAATCAATACCTACCGTCTTTATATAC<br>TTATTAGTCAAGTAGGGGAATAATTTCAGGGAACTGGTTTCAACCTT<br>TTTTTTCAGCTTTTTCCAAATCAGAGAGAGCAGAAGGTAATAGAAGG<br>TGTAAGAAAATGAGATAGATACATGCGTGGGTCAATTGCCTTGTGTC<br>ATCATTTACTCCAGGCAGGTTGCATCACTCCATTGAGGTTGTGCCCGT<br>TTTTTGCCTGTTTGTGCCCCTGTTCTCTGTAGTTGCGCTAAGAGAATG<br>GACCTATGAACTGATGGTTGGTGAAGAAAACAATATTTTGGTGCTGG<br>GATTCTTTTTTTTTCTGGATGCCAGCTTAAAAAGCGGGCTCCATTATA<br>TTTAGTGGATGCCAGGAATAAACTGTTCACCCAGACACCTACGATGT<br>TATATATTCTGTGTAACCCGCCCCCTATTTTGGGCATGTACGGGTTAC<br>AGCAGAATTAAAAGGCTAATTTTTTGACTAAATAAAGTTAGGAAAAT<br>CACTACTATTAATTATTTACGTATTCTTTGAAATGGCAGTATTGATAA<br>TGATAAACTGAGCTCATAGCTTCAAAATGTTTCTACTCCTTTTTTACT<br>CTTCCAGATTTTCTCGGACTCCGCGCATCGCCGTACCACTTCAAAACA<br>CCCAAGCACAGCATACTAAATTTCCCCTCTTTCTTCCTCTAGGGTGTC<br>GTTAATTACCCGTACTAAAGGTTTGGAAAAGAAAAAAGAGACCGCC<br>TCGTTTCTTTTTCTTCGTCGAAAAAGGCAATAAAAATTTTTATCACGT<br>TTCTTTTTCTTGAAAATTTTTTTTTTTGATTTTTTTCTCTTTCGATGACC<br>TCCCATTGATATTTAAGTTAATAAACGGTCTTCAATTTCTCAAGTTTC<br>AGTTTCATTTTTCTTGTTCTATTACAACTTTTTTTACTTCTTGCTCATT<br>AGAAAGAAAGCATAGCAATCTAATCTAAGTTTTAATTACAAA
mhFAS-rTEII	ATGGAAAGATCTGAAGTCGTTATCGCCGGTATGTCTGGTAAATTGCC<br>AGAATCTGAAAACTTGCAAGAATTTTGGGACAACTTGATCGGTGGTG<br>TTGATATGGTTACTGATGATGATAGAAGATGGAAGGCTGGTTTGTAT

	GGTTTGCCAAGAAGATCCGGTAAATTGAAGGATTTGTCCAGATTCGA<br>TGCTTCCTTTTTTGGTGTTCATCCAAAGCAAGCTCATACAATGGACCC<br>ACAATTAAGATTGTTGTTGGAAGTTACCTACGAAGCCATAGTTGATG<br>GTGGTATTAACCCAGATTCTTTGAGAGGTACTCATACTGGTGTTTGG<br>GTTGGTGTTTCTGGTTCTGAAACTTCTGAAGCCTTGTCAAGAGATCCA<br>GAAACTTTGGTTGGTTACTCTATGGTTGGTTGTCAAAGAGCTATGAT<br>GGCTAACAGATTGTCATTCTTCTTCGATTTCCGTGGTCCATCTATTGC<br>TTTAGATACTGCTTGTTCCTCTTCTTTGATGGCCTTGCAAAATGCTTA<br>CCAAGCTATTCATTCTGGTCAATGTCCAGCTGCTATAGTTGGTGGTAT<br>CAATGTTTTGTTGAAGCCAAACACCTCCGTCCAATTTTTGAGATTGGG<br>TATGTTGTCTCCAGAAGGTACTTGTAAGGCTTTTGATACAGCTGGTA<br>ATGGTTATTGCAGATCCGAAGGTGTTGTTGCTGTTTTATTGACCAAAA<br>AGTCCTTGGCCAGAAGAGTTTACGCTACTATTTTGAACGCTGGTACT<br>AATACCGACGGTTTCAAAGAACAAGGTGTTACTTTTCCATCCGGTGA<br>CATCCAAGAACAATTAATCAGATCCTTGTACCAATCTGCTGGTGTTG<br>CTCCTGAATCCTTCGAATATATTGAAGCTCATGGTACTGGTACAAAG<br>GTTGGTGATCCACAAGAATTGAACGGTATTACTAGAGCTTTGTGTGC<br>TACTAGACAAGAACCTTTGTTGATTGGTTCTACCAAGTCTAATATGG<br>GTCATCCAGAACCAGCTTCAGGTTTGGCTGCTTTGGCTAAAGTTTTGT<br>TGTCTTTGGAACATGGTTTGTGGGCTCCAAACTTGCATTTTCATTCTC<br>CAAATCCAGAAATCCCAGCTTTGTTGGATGGTAGATTGCAAGTTGTT<br>GACCAACCATTGCCAGTTAGAGGTGGTAATGTTGGTATCAACTCTTT<br>TGGTTTTGGTGGTTCCAACGTCCACATTATCTTAAGACCAAATACTCA<br>ACCACCACCAGCTCCAGCTCCACATGCTACTTTACCAAGATTATTGA<br>GAGCTTCTGGTAGAACTCCAGAAGCAGTTCAAAAGTTATTGGAACAA<br>GGTTTAAGACACTCCCAAGATTTGGCCTTTTTGTCCATGTTGAATGAT<br>ATTGCTGCTGTTCCAGCTACTGCTATGCCTTTTAGAGGTTATGCTGTT<br>TTGGGTGGTGAAAGAGGTGGTCCAGAAGTTCAACAAGTTCCAGCCG<br>GTGAAAGACCATTGTGGTTTATTTGTTCTGGTATGGGTACTCAATGG<br>AGAGGTATGGGTTTGTCTTTGATGAGATTGGATAGATTCAGAGACTC<br>CATCTTGAGATCAGATGAAGCTGTTAAGCCATTCGGTTTGAAGGTCA<br>GTCAATTATTGTTGTCCACTGACGAATCTACCTTCGATGATATTGTCC<br>ACTCTTTCGTTTCTTTGACCGCCATTCAAATTGGTTTGATCGATTTGTT<br>GTCCTGCATGGGTTTAAGACCAGATGGTATAGTTGGTCATTCTTTGG<br>GTGAAGTTGCTTGTGGTTACGCAGATGGTTGTTTGTCTCAAGAAGAA<br>GCTGTTTTAGCTGCTTATTGGAGAGGTCAATGTATCAAAGAAGCACA<br>TTTGCCACCAGGTGCTATGGCTGCTGTTGGTTTATCTTGGGAAGAATG<br>TAAACAAAGATGTCCACCTGGTGTTGTTCCAGCATGTCATAATTCTA<br>AGGATACCGTTACTATCTCTGGTCCACAAGCTCCAGTTTTCGAATTTG<br>TCGAACAATTAAGAAAAGAAGGTGTTTTCGCCAAAGAAGTTAGAAC<br>TGGTGGTATGGCTTTCCACTCTTACTTTATGGAAGCTATTGCTCCACC<br>ATTATTGCAAGAATTGAAGAAGGTTATCAGAGAACCTAAGCCAAGA<br>TCAGCTAGATGGTTATCTACTTCTATTCCTGAAGCTCAATGGCATTCT<br>AGTTTGGCTAGAACTTCTTCTGCTGAATACAACGTTAACAACTTGGTT<br>TCCCCAGTTTTGTTCCAAGAAGCCTTATGGCATGTTCCAGAACACGCT<br>GTTGTTTTAGAAATTGCTCCTCATGCTTTGTTGCAAGCCGTTTTGAAA<br>AGAGGTTTGAAGCCATCCTGTACCATTATCCCATTGATGAAGAAGGA<br>TCACAGAGACAACTTGGAATTTTTCTTGGCTGGTATCGGTAGATTAC<br>ACTTGTCTGGTATTGATGCTAATCCAAACGCTTTGTTTCCACCAGTTG<br>AATTTCCAGCTCCTAGAGGTACACCATTGATTTCACCTTTGATTAAGT<br>GGGATCATTCCTTGGCTTGGGATGTTCCTGCTGCTGAAGATTTTCCAA

	ATGGTTCTGGTTCACCATCTGCTGCCATATACAATATTGATACCTCTT<br>CCGAATCCCCAGATCACTATTTGGTTGATCATACCTTAGATGGTAGA<br>GTCTTGTTTCCAGCAACTGGTTATTTGTCCATCGTTTGGAAAACATTG<br>GCTAGAGCATTGGGTTTGGGTGTTGAACAATTACCAGTTGTTTTCGA<br>AGATGTCGTCTTGCATCAAGCTACCATTTTGCCAAAAACTGGTACTG<br>TCTCTTTGGAAGTCAGATTATTGGAAGCCTCAAGAGCTTTCGAAGTTT<br>CTGAAAACGGTAACTTGGTTGTTTCCGGTAAAGTTTACCAATGGGAT<br>GATCCAGATCCTAGATTATTTGATCATCCTGAATCTCCAACCCCAAAT<br>CCAACTGAACCATTATTCTTGGCTCAAGCCGAAGTCTACAAAGAATT<br>GAGATTGAGAGGTTACGATTACGGTCCACATTTCCAAGGTATTTTGG<br>AAGCTAGTTTGGAAGGTGACTCTGGTAGATTATTGTGGAAGGATAAT<br>TGGGTCAGTTTCATGGATACCATGTTGCAAATGTCCATTTTGGGTTCT<br>GCTAAACACGGTTTGTATTTGCCAACTAGAGTTACCGCTATTCATATT<br>GATCCAGCTACCCATAGACAAAAGTTGTACACATTGCAAGATAAGGC<br>TCAAGTTGCCGATGTTGTTGTTTCAAGATGGTTGAGAGTTACTGTTGC<br>CGGTGGTGTCCATATTTCTGGTTTACACACTGAATCTGCTCCAAGAA<br>GACAACAAGAACAACAAGTCCCAATTTTGGAAAAGTTCTGTTTCACT<br>CCACATACCGAAGAAGGTTGCTTATCTGAAAGAGCTGCTTTACAAGA<br>AGAATTGCAATTATGCAAGGGTTTGGTTCAAGCCTTGCAAACTAAGG<br>TTACTCAACAAGGTTTGAAAATGGTTGTTCCAGGTTTGGATGGTGCT<br>CAAATTCCTAGAGATCCATCCCAACAAGAATTGCCTAGATTATTGTC<br>TGCTGCTTGCAGATTGCAATTAAATGGTAACTTACAATTAGAATTGG<br>CCCAAGTCTTGGCACAAGAAAGACCAAAATTACCAGAAGATCCATT<br>ATTGTCCGGTTTGTTAGATTCTCCAGCTTTGAAGGCTTGTTTGGATAC<br>TGCTGTTGAAAACATGCCTTCCTTGAAGATGAAGGTTGTTGAAGTTTT<br>GGCTGGTCATGGTCACTTGTATTCAAGAATACCAGGTTTGTTATCCCC<br>ACACCCTTTGTTACAATTATCTTACACTGCCACTGATAGACATCCACA<br>AGCCTTGGAAGCTGCTCAAGCTGAATTGCAACAACATGATGTAGCTC<br>AAGGTCAATGGGACCCAGCTGATCCTGCTCCATCTGCTTTGGGTTCA<br>GCTGATTTGTTGGTATGTAATTGTGCTGTTGCTGCTTTAGGTGATCCA<br>GCATCAGCTTTGTCAAATATGGTTGCTGCATTGAGAGAAGGTGGTTT<br>CTTGTTATTGCACACTTTGTTGAGAGGTCATCCATTGGGTGATATAGT<br>TGCTTTCTTGACTTCTACCGAACCACAATATGGTCAAGGTATATTGTC<br>ACAAGATGCCTGGGAATCTTTGTTCTCAAGAGTCTCTTTGAGATTAGT<br>TGGTTTGAAGAAGTCCTTCTACGGTTCCACTTTGTTTTTGTGTAGAAG<br>ACCAACTCCACAAGATTCCCCAATTTTCTTGCCAGTTGATGACACTTC<br>TTTTAGATGGGTCGAATCCTTGAAGGGTATATTGGCTGATGAAGATT<br>CTTCAAGACCAGTTTGGTTGAAGGCTATTAACTGTGCTACTTCTGGTG<br>TCGTTGGTTTGGTTAACTGTTTGAGAAGAGAACCAGGTGGTAACAGA<br>TTAAGATGCGTCTTATTGTCCAACTTGTCCTCTACTTCTCATGTCCCA<br>GAAGTTGATCCAGGTTCTGCCGAATTGCAAAAAGTTTTACAAGGTGA<br>TTTGGTCATGAACGTCTATAGAGATGGTGCTTGGGGTGCTTTTAGAC<br>ACTTTTTGTTGGAAGAAGATAAGCCTGAAGAACCTACTGCACATGCT<br>TTTGTTTCTACTTTGACTAGAGGTGACTTGTCCAGTATAAGATGGGTT<br>TGCTCATCTTTGAGACATGCTCAACCTACATGTCCAGGTGCTCAATTA<br>TGTACTGTTTACTACGCCTCTTTGAACTTCAGAGATATTATGTTGGCT<br>ACCGGTAAGTTATCTCCAGATGCTATTCCAGGTAAATGGACTTCTCA<br>AGATAGTTTGTTAGGTATGGAATTTTCCGGTAGAGATGCCTCTGGTA<br>AAAGAGTAATGGGTTTAGTTCCAGCAAAAGGTTTGGCAACCTCTGTT<br>TTGTTATCACCAGATTTTTTGTGGGATGTCCCATCTAATTGGACTTTA<br>GAAGAAGCAGCTTCTGTTCCAGTTGTCTATTCTACTGCTTATTACGCT

	TTGGTCGTAAGAGGTAGAGTTAGACCAGGTGAAACCTTATTGATTCA<br>TTCAGGTTCTGGTGGTGTAGGTCAAGCTGCTATTGCTATTGCATTATC<br>TTTGGGTTGTAGAGTATTCACCACTGTTGGTAGTGCTGAAAAAAGAG<br>CTTACTTGCAAGCTAGATTTCCTCAATTAGACTCCACTTCTTTCGCCA<br>ACTCAAGAGATACTTCCTTCGAACAACATGTCTTGTGGCATACCGGT<br>GGTAAAGGTGTTGACTTGGTTTTGAACTCTTTGGCCGAAGAAAAGTT<br>ACAAGCCTCTGTTAGATGTTTGGCTACTCACGGTAGATTCTTGGAAA<br>TCGGTAAGTTTGACTTGTCTCAAAATCACCCTTTGGGTATGGCTATCT<br>TTTTGAAGAACGTTACTTTCCACGGTGTCTTGTTAGATGCTTTCTTCA<br>ATGAATCATCCGCCGATTGGAGAGAAGTTTGGGCTTTAGTTCAAGCT<br>GGTATAAGAGATGGTGTTGTCAGACCATTGAAGTGTACAGTTTTTCA<br>TGGTGCCCAAGTTGAAGATGCCTTTAGATATATGGCACAAGGTAAGC<br>ACATTGGTAAGGTTGTAGTTCAAGTTTTAGCCGAAGAACCTGAAGCA<br>GTTTTGAAAGGTGCTAAGCCAAAATTGATGTCCGCTATTTCTAAGAC<br>TTTCTGCCCAGCTCATAAGTCCTACATTATTGCTGGTGGTTTGGGTGG<br>TTTTGGTTTGGAATTGGCTCAATGGTTGATACAAAGAGGTGTTCAAA<br>AATTAGTCTTGACCTCCAGATCAGGTATTAGAACAGGTTATCAAGCC<br>AAGCAAGTAAGAAGATGGAGAAGACAAGGTGTTCAAGTTCAAGTCT<br>CTACCTCCAATATCTCTTCATTAGAAGGTGCCAGAGGTTTAATTGCTG<br>AAGCTGCACAATTAGGTCCTGTTGGTGGTGTTTTTAACTTGGCCGTTG<br>TTTTGAGAGATGGTTTATTAGAAAATCAAACCCCAGAATTTTTTCAA<br>GACGTCTGCAAGCCTAAATACTCCGGTACTTTGAATTTGGATAGAGT<br>CACTAGAGAAGCCTGTCCAGAATTGGATTACTTCGTTGTTTTCTCCTC<br>TGTTTCTTGTGGTAGAGGTAATGCAGGTCAATCTAATTACGGTTTTGC<br>TAACTCTGCTATGGAAAGAATTTGCGAAAAGAGAAGACACGAAGGT<br>TTGCCAGGTTTAGCTGTTCAATGGGGTGCTATTGGTGATGTCGGTATT<br>TTAGTTGAAACCATGTCTACCAATGACACCATCGTTTCTGGTACTTTG<br>CCACAAAGAATGGCTTCATGTTTGGAAGTATTGGACTTGTTCTTGAA<br>TCAACCACACATGGTTTTGTCCTCATTCGTTTTGGCAGAAAAAGCTGC<br>TGCTTATAGAGATAGAGATTCCCAAAGAGATTTGGTCGAAGCTGTTG<br>CTCATATTTTGGGTATTAGAGACTTAGCTGCCGTTAACTTGGATTCAT<br>CTTTGGCAGATTTGGGTTTAGACTCCTTGATGTCAGTTGAAGTTAGAC<br>AAACTTTGGAAAGAGAATTGAATTTGGTTTTGTCTGTCAGAGAAGTC<br>AGACAATTAACCTTGAGAAAATTACAAGAATTGTCCTCCAAAGCCGA<br>TGAAGCTAGTGAATTGGCTTGTCCTACTCCAAAAGAAGATGGTTTAG<br>CTCAACAACAAACTCAATTAAACGAAACCGCTGTTAATGCCAAGTCT<br>CCTAGAAACGAAAAGGTCTTGAATTGCTTATACCAAAACCCAGATGC<br>CGTTTTCAAGTTGATATGTTTTCCTTGGGCCGGTGGTGGTAGTATTCA<br>TTTTGCTAAATGGGGTCAAAAGATCAACGACTCCTTGGAAGTTCATG<br>CTGTTAGATTAGCTGGTAGAGAAACTAGATTGGGTGAACCATTCGCT<br>AATGACATCTATCAAATTGCCGACGAAATCGTTACTGCCTTGTTGCC<br>AATTATTCAAGACAAGGCCTTTGCCTTTTTCGGTCATAGTTTTGGTTC<br>TTACATTGCCTTGATTACCGCTTTGTTATTGAAAGAAAAGTACAAGA<br>TGGAACCATTGCACATCTTCGTTTCAGGTGCTTCTGCTCCACATAGTA<br>CATCAAGACCACAAGTCCCTGATTTGAATGAATTGACCGAAGAACAA<br>GTAAGACACCACTTGTTGGATTTCGGTGGTACACCAAAACATTTGAT<br>CGAAGATCAAGATGTCTTGAGAATGTTTATCCCTTTATTGAAGGCTG<br>ATGCCGGTGTTGTTAAGAAGTTCATTTTTGACAAACCTTCTAAGGCTT<br>TGTTGTCATTAGACATCACTGGTTTTTTAGGTTCCGAAGATACCATCA<br>AGGATATTGAAGGTTGGCAAGATTTGACCAGTGGTAAATTTGATGTT<br>CACATGTTGCCAGGTGATCACTTCTACTTGATGAAGCCAGATAACGA

	AAACTTCATTAAGAACTACATTGCCAAGTGCTTGGAATTGTCATCAT TGACTCATCATCACCATCACCACTGA

Figure S1

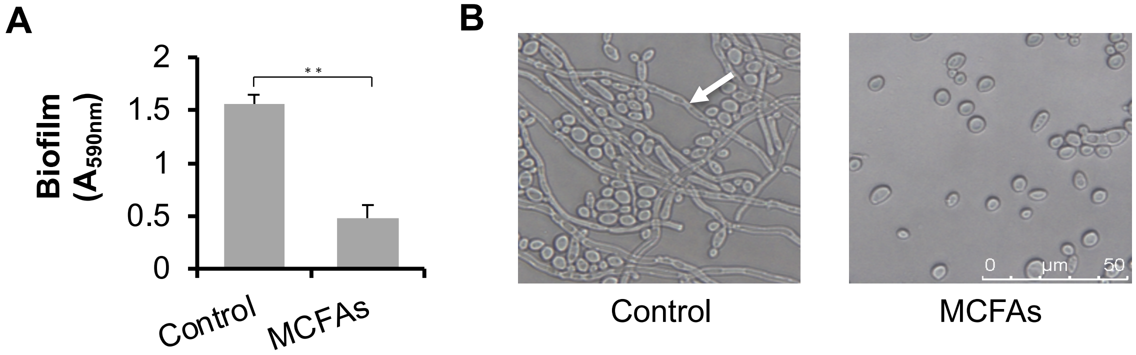

Figure S1. Functional analyses of the commercial MCFAs against biofilm and hyphal formation in *C. albicans* SC5314. A. Effect of the commercial MCFAs on biofilm formation. B. Effect of the commercial MCFA on hyphal formation. Control, without MCFAs. The commercial MCFAs were prepared by mixing respective MCFA (C6:0, C8:0, and C10:0) at the same concentrations as those in the supernatant of the MCFA-producing strains. Hypha is indicated by an arrow. **, $p<0.01$ (Student's t-test).

Figure S2

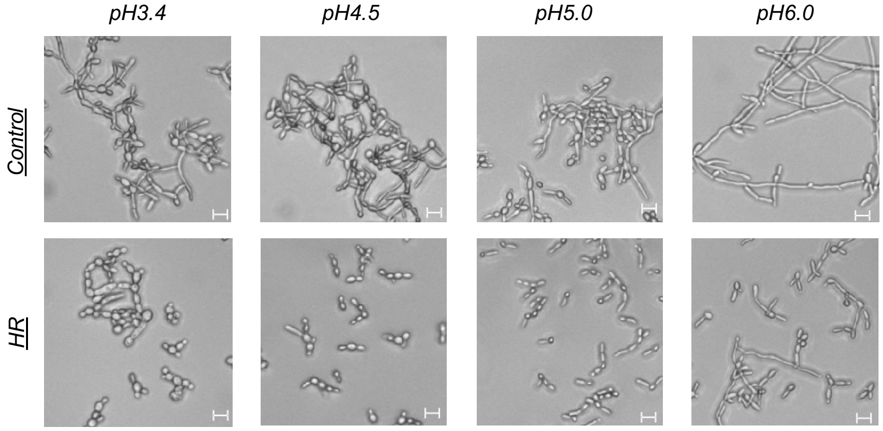

Figure S2. Hyphal formation of *C. albicans 5314* treated by the CLPY04 supernatant (Supernatant HR) and CLPY01 supernatant (Supernatant C) under different pH conditions. Scale bar represents 5 μm.

Figure S3

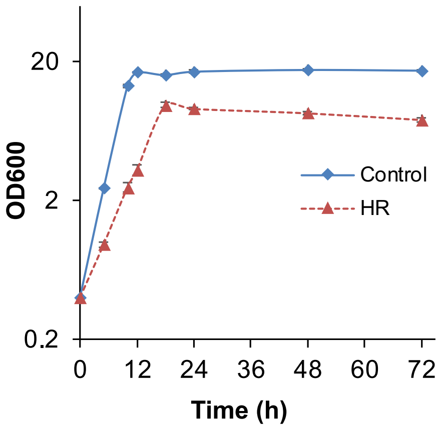

Figure S3. Growth patterns of CLPY04 and CLPY02 in YGD medium containing uracil. Three independent experiments were performed. Control, CLPY02, HR, CLPY04.
